# Factors associated with dehydrating rotavirus diarrhea in children under five in Bangladesh: An urban-rural comparison

**DOI:** 10.1371/journal.pone.0273862

**Published:** 2022-08-26

**Authors:** Sultana Yeasmin, S. M. Tafsir Hasan, Mohammod Jobayer Chisti, Md Alfazal Khan, A. S. G. Faruque, Tahmeed Ahmed

**Affiliations:** 1 Nutrition and Clinical Services Division, International Centre for Diarrhoeal Disease Research, Bangladesh (icddr,b), Dhaka, Bangladesh; 2 Health System and Population Studies Division, International Centre for Diarrhoeal Disease Research, Bangladesh (icddr,b), Dhaka, Bangladesh; 3 Office of the Executive Director, International Centre for Diarrhoeal Disease Research, Bangladesh (icddr,b), Dhaka, Bangladesh; University of Illinois at Chicago, UNITED STATES

## Abstract

**Introduction:**

Rotavirus is the leading cause of dehydrating diarrhea in young children worldwide. This study aimed to identify the factors associated with dehydrating rotavirus diarrhea in children under five years of age in urban and rural Bangladesh.

**Methods:**

The study analyzed data from 7,758 children under five who presented with rotavirus diarrhea to Dhaka (urban) and Matlab (rural) hospital of icddr,b during 2009–2018, and were enrolled in the Diarrheal Disease Surveillance System. Cases were defined as children having rotavirus isolated in stool specimens presented with dehydrating diarrhea. Controls were children infected with rotavirus have no dehydration. Multivariable logistic regression models were built to identify the factors associated with dehydrating diarrhea.

**Results:**

Among the rotavirus-infected children, 1,784 (34%) in Dhaka and 160 (6%) in Matlab had diarrhea with some or severe dehydration. The female children and age group 24–59 months age was found to be at higher risk of dehydration compared to 6–11 months age. In the multivariable logistic regression model, maternal illiteracy, vomiting, the onset of diarrhea less than 24 hours prior to presenting to the hospital, monsoon months, stunting, and wasting were significantly associated with dehydrating rotavirus diarrhea among children aged 0–59 months in Dhaka. In Matlab, monthly income, duration less than 24 hours prior to attending the hospital, and wasting had an independent significant association with dehydrating rotavirus diarrhea episodes.

**Conclusions:**

Considering factors diversity, educating parents and proper counselling by health care personnel during diarrhea, could lessen the severity of dehydration and the number of hospital visits later on by eliminating the modifiable risk factors among the children, which needs further studies.

## Introduction

Diarrhea is the major cause of clinical illnesses and mortality in children younger than five years of age. Globally, diarrhea accounts for 8% of 5.2 million deaths as reported in 2019 [1]. Among children younger than five years, the number of annual diarrheal episodes and deaths are significantly higher in South East Asia and Sub- Saharan Africa compared to developed countries [2]. In India, diarrhea causes 55,309 deaths in children under 5 as reported in 2019 [3]. Clinically severe diarrhea manifested by dehydration is mainly responsible for high case fatality [4]. Rotavirus is one of the important pathogens causing dehydrating diarrhea and hospitalization in children less than 5 years of age [5,6]. Every year about 260 million diarrheal episodes occur in infants and children below 5 years old due to rotavirus infection worldwide and of them, more than 1.5 million cases become severe enough to be admitted to the hospital despite the introduction of the rotavirus vaccine in several countries across the globe [7,8]. The proportion of children under five hospitalized with rotavirus diarrhea has increased from 20% in 1993 to 64% in 2015 [9,10]. Other enteroviruses including adenovirus and norovirus are becoming emerging infectious agents and are observed to be associated with a growing number of deaths compared to rotaviral diarrhea in the recent past [11,12]. Yet the proportion of deaths from rotavirus diarrhea in children is concerning. Prevention of newer episodes of rotavirus diarrhea by rota vaccination is evident but the long-term impact of rota vaccine on the severity of diarrhea is poorly understood [13–15]. Studies in rural, Bangladesh reported Pentavalent Rota Vaccine (PRV) had moderate efficacy in infants [16] and indirectly associated with less number of rotavirus diarrhea patients presented in treatment facilities [17]. Due to the evolving disease epidemiology and heterogeneity of childhood diarrhea, underscores the need for further investigation to explore the factors associated with the progression of dehydrating rotavirus diarrhea in children younger than 5 years [18]. Nevertheless, there is a paucity of information on the underlying factors associated with dehydrating rotavirus diarrhea [19] and whether these factors differ between urban and rural children. We aimed to investigate the clinical, sociodemographic, nutritional, and environmental factors associated with dehydrating rotavirus diarrhea in children under five, living in urban and rural Bangladesh.

### Study settings

Established by icddr,b back in 1961, the Dhaka Hospital is located in urban Dhaka, the capital city of Bangladesh. The hospital provides free-of-cost treatment to over 150,000 diarrheal patients each year. The hospital has maintained a diarrheal disease surveillance system (DDSS) since 1979 and currently samples 2% (every 50^th^) of patients by systematic sampling for participation. Extensive microbiological assessments of fecal samples are performed to identify diarrheal pathogens and antimicrobial susceptibility of common bacterial enteric pathogens.

The Matlab Hospital is located in rural Matlab, about 55 kilometers southeast of Dhaka, and provides free-of-cost treatment to over 40,000 diarrheal patients annually. Although the hospital has been treating diarrhea patients since 1963, DDSS was extended to Matlab in 1999. In Matlab, DDSS enrolls all patients coming from the area covered by the Health and Demographic Surveillance System (HDSS) of icddr,b. The detailed discretions of Dhaka and Matlab hospitals, DDSS, and HDSS has been provided elsewhere [20–22].

### Study design, population, and data source

We used an unmatched case-control design for this study. Children <5 years of age with rotavirus mono-infection and clinical dehydration constituted the cases. Children under five with rotavirus isolated from their stools but without clinical dehydration constituted the controls. Our analyses excluded children with co-infections. Trained study personnel collected relevant data, on socio-demographics, household characteristics, and feeding practices, particularly of infants and young children. Additionally, information was gathered regarding the use of drugs and fluid therapy at home by interviewing parents or caregivers using structured questions. They also collected anthropometric measurements and recorded the findings related to the nutritional status of children. A clinician evaluated clinical features, recorded findings after physical examination, provided all needed treatments, and recorded the outcomes of patients.

All study-related data were retrieved from the electronic database of DDSS. The stool samples were tested for rotavirus in the Virology Laboratory of icddr,b in Dhaka then test results were included in the database, and later on, provided to the authors for analysis.

### Definitions

Diarrhea was defined as the passage of three or more abnormally loose stools per day. The severity of dehydration was defined as the condition that results from excessive loss of body water and electrolytes [23]. Clinical dehydration was defined as having some or severe dehydration. Classification of some and severe dehydration was done following icddr,b’s assessment method, which was also recommended by the World Health Organization (WHO) [24]. Explicitly, diarrhea patients presented with at least two of these clinical signs (restless, sunken eyes, thirsty and drinks eagerly, and skin pinch goes back slowly) were defined as some dehydration. Severe dehydration was graded with the presence of two or more of the following signs (lethargic or unconscious, sunken eyes, drinks poorly or not able to drink, skin pinch goes back very slowly). Diarrhea children not fulfilling the criteria of some or severe dehydration were remarked as having no dehydration (The treatment of diarrhea, A manual for physicians and other senior health workers, WHO, 2005). The classification of underweight, wasting, and stunting was done by Weight-for-age Z-score(WAZ), weight-for-height Z-score (WHZ), and Length/height-for-age Z-score less than two standard deviations below the median of WHO growth standards, respectively [25]. An axillary temperature of ≥37.8°C indicated a fever [26]. Bangladesh has three distinct seasons: the hot summer season from March to June; the rainy monsoon season from July to September; and the dry winter season from October to February [27].

### Laboratory methods

After collection, stool specimens were divided and submitted to relevant icddr,b laboratories for culture and enzyme-linked immunosorbent assay (ELISA) [16,28]. Rotavirus and other enteric pathogens, including *V*. *cholerae*, *Shigella*, *Salmonella*, *Amoeba*, *and Giardia* species were isolated and characterized using standard laboratory methods in the Clinical Microbiology Laboratory [29,30]. The commercially available ProSpect rotavirus kit (Catalog No. R240396, Oxoid Ltd., Basingstoke Hants, UK), is a qualitative enzyme immunoassay which utilizes a polyclonal antibody in a solid phase sandwich-type enzyme immunoassay was used to detect group-specific antigen present in Group A rotavirus in align with manufacturer’s instruction [31,32].

### Data analysis

We presented the descriptive characteristics of the study population using proportion. To identify the factors associated with dehydrating rotavirus diarrhea, simple and multiple logistic regression analysis were performed. Strength of association was expressed as odds ratios (ORs) with their 95% confidence intervals (CIs). Variables with a p-value less than 0.2 in the bivariate model were initially considered for multivariable logistic regression model building [33]. However, only the significant variables were retained in the final model. A p-value less than 0.05 was considered statistically significant. Yearly exchange rates of US dollars (USD) were used to estimate monthly family income in USD. Length/height-for-age Z-score and weight-for-length/height Z-score were calculated by the WHO Anthro (version 3.2.2; Department of Nutrition, WHO, Geneva, Switzerland). The rest of the data analysis was done using Stata/PC (version 15.1; Stata Corp).

### Ethical considerations

The collection of information via DDSS was approved by the Research Review Committee (RRC) and the Ethical Review Committee (ERC) of icddr,b. At the time of enrollment, verbal consent was obtained from caregivers or guardians, documented on the questionnaire, and shown to the consenting party. Parents and guardians were assured about the nondisclosure of information collected from them, were informed about the use of data for analysis, and icddr,b’s plan to use the results for improving patient care activities, including publication of de-linked data. The information was stored in the DDSS’s electronic database. The ERC of icddr,b accepted the verbal consenting procedure and approved the collection of data under DDSS.

## Results

Between 2009 and 2018, DDSS enrolled 14,234 patients under the age of five in Dhaka and 6,779 in Matlab. Rotavirus was isolated from 5,250 and 2,508 of the children younger than five years in Dhaka and Matlab, respectively. Among the rotavirus-infected children, 1,784 (34%) in Dhaka and 160 (6%) in Matlab had some or severe dehydration.

In bivariate analysis use of the non-sanitary toilet, monthly income ≤100 USD, living in a slum, duration of diarrhea <1 day, and not using oral rehydration solution (ORS) at home before reporting to the hospital and monsoon season were associated with dehydrating rotavirus diarrhea in Dhaka (Table 1). The children in age groups 0–5 months and 24–59 months were found to be associated with dehydrating diarrhea compared to 6–23 months. The children with dehydrating rotavirus diarrhea more often had a clinical manifestation of fever and vomiting compared to those who had no dehydration. The stunted and wasted children had a higher association with developing dehydrating rotavirus diarrhea than their counterparts (Table 1).

**Table 1:** Characteristics of rotavirus-infected under-five children with (cases) or without (controls) clinical dehydration in Dhaka.

	Cases (1784), n (%)	Controls (3466), n (%)	OR^1^	95% CI	P
Demographics					
Age in months, 0–5	265 (14.9)	444 (12.8)	1.3	1.1–1.5	0.007
Age in months, 6–23	1375 (77.0)	2891 (83.4)	-	-	-
Age in months, 24–59	144 (8.1)	131 (3.8)	2.3	1.8–3.0	<0.001
Female sex	699 (39.2)	1258 (36.3)	1.1	1.01–1.3	0.041
Illiterate mother	251 (14.1)	316 (9.1)	1.6	1.4–1.9	<0.001
Use of non-sanitary latrine	322 (18.1)	542 (15.6)	1.2	1.02–1.4	0.026
Monthly income ≤100 USD	410 (23.0)	649 (18.7)	1.3	1.1–1.5	<0.001
Distance traveled >5 miles	1650 (92.5)	3166 (91.3)	1.2	0.9–1.4	0.154
Living in slums	65 (3.6)	83 (2.4)	1.5	1.1–2.1	0.010
Symptoms					
Vomiting	1467 (82.2)	2548 (73.5)	1.7	1.4–1.9	<0.001
Fever	125 (7.0)	192 (5.5)	1.3	1.02–1.6	0.035
Pre-hospital diarrhea <1 day	492 (27.6)	781 (22.5)	1.3	1.1–1.5	<0.001
Home treatment					
No ORS at home	50 (2.8)	60 (1.7)	1.6	1.1–2.4	0.010
Season					
Summer	413 (23.2)	781 (22.5)	1.1	1.0–1.3	0.116
Monsoon	319 (17.9)	459 (13.2)	1.5	1.3–1.7	<0.001
Nutritional status					
Stunting	380 (21.9)	590 (17.4)	1.3	1.2–1.5	<0.001
Wasting	438 (25.3)	432 (12.7)	2.3	2.0–2.7	<0.001

^1^ORs were calculated for the presence of clinical dehydration (case) compared to no dehydration (control).

Reference categories: Age 6–23 months, male sex, literate mother, use of sanitary latrine, monthly income >100 USD, distance traveled ≤5 miles, not living in slums, no vomiting, normal temperature, duration of diarrhea ≥1 day before arrival at icddr,b hospital, use of ORS at home, winter season, no stunting, and no wasting.

Abbreviations: OR, odds ratio; CI, confidence interval; USD, United States Dollar; ORS, oral rehydration solution.

Findings from the bivariate analysis showed in Matlab cases coming from the family using a non-sanitary latrine, monthly income ≤100 USD, duration of diarrhea < 1day before reporting in hospital, those who were stunted and wasted had more often dehydrating rotavirus diarrhea compared with the children with no stunting or wasting. Those children younger than five years of age not receiving ORS at home, having fever, and vomiting were not associated with clinical dehydration in rotavirus-infected children (Table 2).

**Table 2:** Characteristics of rotavirus-infected under-five children with (cases) or without (controls) clinical dehydration in Matlab.

	Cases (166), n (%)	Controls (2413), n (%)	OR^1^	95% CI	P
Demographics					
Age in months, 0–5	16 (10.0)	205 (8.7)	1.2	0.7–2.0	0.580
Age in months, 6–23	132 (82.5)	1969 (83.9)	-	-	-
Age in months, 24–59	12 (7.5)	174 (7.4)	1.0	0.6–1.9	0.928
Female sex	58 (36.3)	859 (36.6)	1.0	0.7–1.4	0.932
Illiterate mother	7 (4.4)	87 (3.7)	1.2	0.5–2.6	0.666
Use of non-sanitary latrine	133 (83.1)	1687 (71.9)	1.9	1.3–3.0	0.002
Monthly income ≤100 USD	77 (48.1)	618 (26.3)	2.6	1.9–3.6	<0.001
Distance traveled>5 miles	70 (43.8)	943 (40.2)	1.1	0.8–1.6	0.371
Symptoms					
Vomiting	140 (87.5)	1956 (83.3)	1.4	0.9–2.3	0.166
Fever	28 (17.5)	349 (14.9)	1.2	0.8–1.9	0.367
Pre-hospital diarrhea <1 day	75 (46.9)	772 (32.9)	1.8	1.3–2.5	<0.001
Home treatment					
No ORS at home	20 (12.5)	258 (11.0)	1.2	0.7–1.9	0.556
Season					
Summer	30 (18.8)	470 (20.0)	0.9	0.6–1.4	0.706
Winter	102 (63.7)	1474 (62.8)	-	-	-
Monsoon	28 (17.5)	404 (17.2)	1.0	0.6–1.5	0.994
Nutritional status					
Stunting	42 (26.3)	453 (19.4)	1.5	1.03–2.1	0.035
Wasting	40 (25.2)	269 (11.5)	2.6	1.8–3.8	<0.001

^1^ORs were calculated for the presence of clinical dehydration (case) compared to no dehydration (control).

Reference categories: Age 6–23 months, male sex, literate mother, use of sanitary latrine, monthly income >100 USD, distance traveled ≤5 miles, no vomiting, normal temperature, duration of diarrhea ≥1 day before arrival at icddr,b hospital, use of ORS at home, winter season, no stunting, and no wasting.

Abbreviations: OR, odds ratio; CI, confidence interval; USD, United States Dollar; ORS, oral rehydration solution.

At multivariate logistic regression analysis, for children visiting Dhaka Hospital, dehydrating rotavirus diarrhea was significantly associated with maternal illiteracy, diarrhea duration of less than 24 hours before arriving at the hospital, vomiting, fever, stunting and wasting. While compared with 6–23 months old children the increased risk of developing dehydration was associated with younger children (0–5 months) and this risk was, even more in older (24–59 months) children. Female children were more likely than males to have dehydrating diarrhea. Wasting and stunting were associated with clinical dehydration, but being underweight was not. In the multivariable model in Dhaka, participants who traveled a distance over 5 miles to arrive in the hospital were also associated with a higher risk of dehydration than those who travelled shorter distance (Table 3).

**Table 3:** Results of multiple logistic regression to explore the independent predictors of clinical dehydration among rotavirus-infected under-five children in Dhaka.

Characteristics	AOR^1^	95% CI	P
Age in months, 0–5	1.2	1.004–1.4	0.045
Age in months, 24–59	2.1	1.6–2.7	<0.001
Female sex	1.2	1.03–1.3	0.015
Illiterate mother	1.5	1.2–1.8	<0.001
Distance traveled>5 miles	1.3	1.02–1.6	0.033
Vomiting	1.7	1.5–2.0	<0.001
Fever	1.3	1.004–1.6	0.046
Pre-hospital diarrhea <1 day	1.3	1.1–1.4	0.001
Summer season	1.1	0.9–1.3	0.272
Monsoon season	1.4	1.2–1.7	<0.001
Stunting	1.3	1.1–1.5	0.002
Wasting	2.2	1.9–2.5	<0.001

^1^Adjusted OR from a multivariable model that includes age, sex, mother’s literacy, distance traveled to arrive at icddr,b hospital, presence of vomiting and fever, duration of diarrhea before arrival at icddr,b hospital, season, stunting, and wasting.

Reference categories: Age 6–23 months, male sex, literate mother, distance traveled ≤5 miles, no vomiting, normal temperature, duration of diarrhea ≥1 day before arrival at icddr,b hospital, winter season, no stunting, and no wasting.

Abbreviations: AOR, adjusted odds ratio; CI, confidence interval.

The rotavirus isolation was relatively low, and the proportion of cases with clinical dehydration was relatively higher during the monsoon months in both Dhaka and Matlab.

As per multivariable logistic regression, analysis of rotavirus-infected children attending the Matlab Hospital were more likely to experience clinical dehydration if they had a monthly family income of ≤USD 100 and experienced diarrhea continuing for less than 24 hours before seeking care from the hospital. Wasting was significantly associated with dehydrating rotavirus diarrheal episodes, but stunting was not, unlike children from Dhaka Hospital (Table 4).

**Table 4:** Results of multiple logistic regression to explore the independent predictors of clinical dehydration among rotavirus-infected under-five children in Matlab.

Characteristics	AOR^1^	95% CI	P
Monthly income ≤100 USD	2.3	1.6–3.2	<0.001
Pre-hospital diarrhea <1 day	1.7	1.2–2.4	0.002
Wasting	2.1	1.5–3.2	<0.001

^1^Adjusted OR from a multivariable model that includes monthly family income, duration of diarrhea before arrival at icddr,b hospital, and wasting.

Reference categories: Monthly income >100 USD, duration of diarrhea ≥1 day before arrival at icddr,b hospital, and no wasting.

Abbreviations: AOR, adjusted odds ratio; CI, confidence interval; USD, United States Dollar.

## Discussion

This study identified that dehydrating rotavirus diarrhea is a substantial disease burden in children 0–59 months of age and the proportion of some or severe dehydration is higher in urban children compared to rural children in Bangladesh. Likewise, the GEMS (Global Enteric Multicenter Study) study identified rotavirus as the most attributable causative organism for dehydrating diarrhea in children in resource constraint settings [5,34]. The variability of the prevalence of rotavirus diarrhea was found to be associated with sociodemographic status, seasonality, location of residence, source of drinking water, and strain differences of rotavirus [35,36]. Anecdotal data also supported an association between rotavirus diarrhea and significant growth faltering in children less than five years mostly in developing countries [37].

In the present study, many of the factors were observed to be associated with dehydrating rotavirus diarrheal episodes in under 5 children with diarrheal illness in Dhaka. Most notably, children under five infected with rotavirus were more likely to experience dehydration if they had an illiterate mother, onset of diarrhea less than 1 day prior to the hospital visit, traveling a distance of more than 5 miles, vomiting, fever, stunted and wasted children.

Only a limited number of factors associated with clinical dehydration were common to both Dhaka and Matlab, in cases of a particular duration of diarrhea less than 1 day before reporting to hospital and wasting.

Maternal illiteracy, an indicator of worsening childcare, is associated with major childhood infections, including diarrhea and its consequences like malnutrition [38–40]. Similarly, loss of appetite, vomiting, and fever are important contributors to dehydration in diarrheal children regardless of etiology [41]. These may lead to early-onset (less than 24 hours) of dehydration among the rotavirus gastroenteritis children, as suggested by our study findings [42]. The presence of higher episodes of diarrhea and frequent vomiting during the first 24 hours [43], fever, and less intake of ORS might have caused children to develop dehydration [44,45] and make the children sicker. So, caregivers sought care early for this group of children having rotaviral diarrhea and dehydration. Other studies also found that parents seek prompt care from hospitals when they perceive rapidly worsening clinical conditions for severe dehydration [46]. However, as no invasive testing was done, we are unable to postulate any relationship between the pathophysiology of rotavirus diarrhea, dehydration, and the shorter duration of pre-hospital diarrhea. In Bangladesh and elsewhere, rotavirus diarrhea occurs throughout the year, but the incidence and prevalence are much higher during the winter months [27]. In our study, the proportion of clinical dehydration was relatively higher during the monsoon and lesser during the winter season. This could be due to seasonal variation in the rotavirus serotype [47]. Serotypes G9 and G4 were found to be associated with an increased risk of dehydration and G4 is predominant from September to November [48,49]. Young infants (less than 6 months) were twenty percent more at risk of developing dehydration but the risk of developing dehydration among older children (24–59 months age) was more than double compared to 6–23 months old children. A similar trend was observed in bivariate analysis in Matlab but the difference was not statistically significant which might be due to the small sample size. In Bangladesh, breastfeeding is more common in the first 6 months of age, which might have an impact on the severity of dehydration. A study in Matlab reported that breastfed babies are less likely to develop severe dehydration [50,51]. Overall, the number of children with rotavirus diarrhea and the proportion with dehydration was lower in Matlab than in Dhaka. Children in Matlab could have been protected from rotavirus infections, as well as less-severe diseases along with immediate administration of oral rehydration therapy at home, quantification of ORS or home-based fluids consumed at home could better elucidate this finding. A clinical trial was conducted between 2007 and 2009 that studied the efficacy of a Pentavalent Rotavirus Vaccine (PRV); accordingly, the trial may have improved herd immunity and increased serum anti-rotavirus IgA responses in this population [52]. The virulence and strain difference in these two sites could be other contributing factors [53,54]. Rotavirus infection has a relationship with nutritional status: nutritionally healthier children have been observed to be at lower risk of rotavirus infection, and overweight and obese children have been observed to be at higher risk of rotavirus diarrhea than severely malnourished children [55,56]. We noted rotavirus-infected malnourished (wasted) children to be at a higher risk of dehydrating diarrhea than their nutritionally better-off counterparts. In previous studies, acutely malnourished children experienced severe dehydration as a result of vomiting, fever, and profuse volume of watery stool, the latter results from a consequence of extensive gut barrier dysfunction as revealed [57,58]. In the present study, in Dhaka, children with rotavirus gastroenteritis were more likely to have clinical dehydration than Matlab. Such explanation may include environmental contamination with prevailing poor water and sanitation systems and consequent higher infective dose (large inoculums size) [59], co-morbidity, poor parental literacy [60], lack of maternal knowledge on preparation and use of ORS, and engagement of mothers in out of home activities leading to less caring of their children during illness [61]. The slum children in Dhaka were also more malnourished and less immunized, and other studies have found they often suffer from micronutrient and vitamin A deficiencies [62], all of which may exacerbate dehydrating diarrheas. A recent study on school-aged children found that children with vitamin A deficiency were at double the risk of diarrhea with vomiting than children with adequate vitamin A status [63]. Zodopey et al. observed an association between dehydrating diarrhea and receipt of ORS at home [41] which is not consistent with our study findings. This discrepancy is likely because ORS influences the severity of diarrhea and care-seeking behavior cyclically–children with more severe diarrhea are more likely to receive ORS and more likely to seek out medical care and thus become less dehydrated [64]. In another study, Taylor et al. noted the less frequent success of oral rehydration therapy (ORT) in children <5 years of age with more severe diarrhea [22]. Although bacterial diarrhea was more frequently responsible for dehydration and use of IV fluid [65], our study showed that a considerable proportion of children under five with rotavirus gastroenteritis was also dehydrated and required IV fluid, which is consistent with other studies [66]. In our study, we noted several characteristics among children with rotavirus diarrhea that were associated with clinical dehydration. An early observation of the aforementioned factors may help to identify children at higher risk of clinical dehydration and allow caregivers and medical professionals to take measures to prevent dehydration, which reduces hospitalization, increases efficiency, and reduces treatment costs [67]. We used routine surveillance data where only rotavirus was screened out except for other enteric viral pathogens and thus could not rule out any effect of other viral agents as mixed infection of dehydrating diarrhea. However, by eliminating fecal co- pathogens have lessened the influence of mixed infection as comorbidity. Nevertheless, a stringent selection of cases and controls with rotavirus mono-infection instead of healthy controls produced results specifically associated with rotavirus-induced dehydration.

## Conclusions

We observed significant differences in gender of the child, maternal literacy, vomiting, fever, duration of diarrhea before seeking care, intake of oral rehydration solution, and nutritional status differentials between dehydrating and non-dehydrating rotavirus diarrhea in children younger than 5 years of age, in both urban and in rural areas. Our study participants were children under five years of age seeking care from facilities that may not be representative of the general population. However, Systematic sampling and unbiased enrollment, large data sets from both sites, and high-quality laboratory procedures are the strengths of our analysis. Early identification of children at higher risk for dehydration may help clinicians and caretakers to take appropriate measures at the early stages of the illness. This finding can help policymakers to determine efficient ways to prevent dehydrating diarrheal episodes. As suggested by contextualizing our results within the broader literature, introducing the rotavirus vaccine in routine immunization programs in concert with other preventive measures, including promotion of breastfeeding and health education to expand awareness of caregivers, may help reduce childhood dehydrated diarrheal disease burden of children under 5 living in remote resource constraint as well as underprivileged settings. Strengthening preventive measures along with rotavirus vaccine coverage and implementing the diarrhea management guideline prioritizing high-risk children through the health systems of the country could reduce the burden of severe rotavirus diarrhea.
